# Intensification of the ultrafiltration of real oil-contaminated (produced) water with pre-ozonation and/or with TiO_2_, TiO_2_/CNT nanomaterial-coated membrane surfaces

**DOI:** 10.1007/s11356-020-08047-1

**Published:** 2020-02-14

**Authors:** Gábor Veréb, Péter Kassai, Erika Nascimben Santos, Gangasalam Arthanareeswaran, Cecilia Hodúr, Zsuzsanna László

**Affiliations:** 1 Institute of Process Engineering, Faculty of Engineering, University of Szeged, Moszkvai Blvd. 9., Szeged, HU-6725 Hungary; 2 Membrane Research Laboratory, Department of Chemical Engineering, National Institute of Technology, Tiruchirappalli, Tamilnadu 620015 India; 3 Institute of Environmental Science and Technology, University of Szeged, Tisza Lajos Blvd. 103, Szeged, H-6720 Hungary

**Keywords:** Oil, Produced water, Membrane filtration, Ultrafiltration, Combination, Pre-ozonation, Modified membrane, Nanomaterial

## Abstract

In the present study, commercial PES, PVDF, PTFE ultrafilter membranes, and two different nanomaterial (TiO_2_ and TiO_2_/CNT composite)-covered PVDF ultrafilter membranes (MWCO = 100 kDa) were used for the purification of an industrial oil-contaminated (produced) wastewater, with and without ozone pretreatment to compare the achievable fouling mitigations by the mentioned surface modifications and/or pre-ozonation. Fluxes, filtration resistances, foulings, and purification efficiencies were compared in detail. Pre-ozonation was able to reduce the total filtration resistance in all cases (up to 50%), independently from the membrane material. During the application of nanomaterial-modified membranes were by far the lowest filtration resistances measured, and in these cases, pre-ozonation resulted in a slight further reduction (11–13%) of the total filtration resistance. The oil removal efficiency was 83–91% in the case of commercial membranes and > 98% in the case of modified membranes. Moreover, the highest fluxes (301–362 L m^−2^ h^−1^) were also measured in the case of modified membranes. Overall, the utilization of nanomaterial-modified membranes was more beneficial than pre-ozonation, but with the combination of these methods, slightly higher fluxes, lower filtration resistances, and better antifouling properties were achieved; however, pre-ozonation slightly decreased the oil removal efficiency.

## Introduction

In the last few decades, water pollution has become a significant global problem due to the rapidly growing population and industrialization (Zhang et al. 2018). Oily wastewaters are produced by several industries, and oil discharges are harmful to the natural environment, which cause ecology problems and endanger human health directly/indirectly through the food chain (Boleydei et al. 2018; Yang et al. 2019; Yu et al. 2015). Therefore, efficient elimination of oily pollutants is of utmost importance both from environmental and human health aspects. Accordingly, the development of efficient methods to treat oily wastewater has great interest, and the widespread application of such effective treatments is expected in the future (Cai et al. 2018; Wu et al. 2018; Yang et al. 2019; Yu et al. 2015). To meet the stringent emission limits, combined methods must be used, including one or more conventional technique(s) such as skimming (Stewart and Arnold 2009), sand filtration (Hong and Xiao 2013; Zaneti et al. 2011), centrifugation (Cambiella et al. 2006), flotation (Al-Shamrani et al. 2002; Rubio et al. 2002), adsorption (Boleydei et al. 2018), or chemical destabilization (Rodriguez Boluarte et al. 2016; Zaneti et al. 2011; Zolfaghari et al. 2016), augmented with advanced oxidation processes (Hong and Xiao 2013; Rodriguez Boluarte et al. 2016) and/or membrane filtration (Fakhru'l-Razi et al. 2009; Matos et al. 2016; Padaki et al. 2015). This enables the effective elimination of not just the floating and dispersed oil but the emulsified micro- and nano-sized oil droplets as well. As membrane filtration, microfiltration (Abadi et al. 2011; Hu et al. 2015; Masoudnia et al. 2014; Salahi et al. 2010; Shokrkar et al. 2012; Zhang et al. 2018), ultrafiltration (Masoudnia et al. 2014; Saki and Uzal 2018; Salahi et al. 2010; Yi et al. 2011), nanofiltration (Golpour and Pakizeh 2017; Zhao et al. 2019), or even reverse osmosis (Kasemset et al. 2013) can be used, resulting in increasing purification efficiency in this order.

Membrane separation techniques are widely used in wastewater treatment since they can be easily scaled up and integrated; moreover, they have low operating cost and high removal efficiency (Golpour and Pakizeh 2017). However, fouling has been a crucial challenge of membrane filtration processes since the birth of this technology, especially in the case of oil-in-water emulsions. Oily contaminants quickly form a hydrophobic layer acting as a significant water barrier on the membrane surface, which reduces water flux, decreases membrane lifespan, and increases energy consumption (Liu et al. 2018; Matos et al. 2016; Padaki et al. 2015; Yin and Zhou 2015; Zhao et al. 2019).

Various strategies have been investigated to mitigate membrane fouling such as the improvement of operational conditions, feed pretreatment methods, and membrane modification processes. Among the suitable pretreatment methods, chemical destabilization (Matos et al. 2016; Metcalfe et al. 2016), ion exchange (Lindau and Jijnsson 1994), gas injection (Um et al. 2001), and pre-oxidation (Xue et al. 2016) proved to be efficient to reduce the accumulation of the oily contaminants on membrane surfaces. For the membrane filtration of wastewaters contaminated with antibiotics (Alpatova et al. 2013), humic acid (Byun et al. 2015; Jermann et al. 2008), or natural organic matter (Cheng et al. 2016), pre-ozonation was found to be a beneficial pretreatment. Recently, it was also proved to be efficient to decrease membrane fouling in the case of oily wastewaters (Veréb et al. 2018b; Xue et al. 2016) due to the effective surface charge modification of the oil droplets. This results in reduced adherence ability on the membrane surface because of the increased electrostatic repulsive force between the oil droplets and the membrane material.

Membrane surface modification – via the improvement of the hydrophilicity – is another very effective method to reduce membrane fouling in the case of hydrophobic oily contaminants. There are various methods to improve the hydrophilicity of membranes (Miller et al. 2017; Van der Bruggen 2009) including sulfonation (Baroña et al. 2007), carboxylation (Sajitha et al. 2002), polymer blending (Fang et al. 2017), plasma- or UV-induced grafting (Susanto et al. 2007; Wavhal and Fisher 2002; Wu et al. 2018), and nanoparticle-based surface modifications (Hu et al. 2015; Islam et al. 2017; Saki and Uzal 2018; Yi et al. 2011; Yin and Zhou 2015; Zhou et al. 2010). Among the various nanomaterials, the ones with photocatalytic properties can provide – apart from the increased hydrophilicity – the possibility to degrade organic fouling contaminants by simple UV or solar irradiation, which proved to be efficient to decompose oily contaminants from membrane surfaces (Chang et al. 2014; Gondal et al. 2014; Moslehyani et al. 2015; Pan et al. 2012; Shi et al. 2016; Tan et al. 2015). In our recent studies (Kovács et al. 2018; Veréb et al. 2018a), TiO_2_ and TiO_2_/CNT coatings resulted in excellent antifouling effect during the ultrafiltration of synthetic oil-in-water emulsions and, in addition, TiO_2_/CNT-composite-modified membrane showed increased photocatalytic activity.

In the present study, two recently published and very promising methods, namely, the nanomaterial-based modification of membranes (Veréb et al. 2018a) and/or pre-ozonation (Veréb et al. 2018b; Veréb et al. 2019) were used separately and simultaneously for the mitigation of fouling during the ultrafiltration of an industrial oil-contaminated (produced) water. Investigated membrane materials widely used were polyethersulfone (PES), outstandingly durable polytetrafluoroethylene (PTFE), and polyvinylidene fluoride (PVDF), which is widely used to prepare nanomaterial-modified membranes. Achievable fluxes, filtration resistances, fouling mechanisms, and purification efficiencies were compared in detail in the case of the different membranes, with or without the application of a short pre-ozonation to determine the achievable advantages in a realistic system.

## Materials and methods

### Description of the collected produced water

The investigated oil-contaminated wastewater (produced water) was provided by a South Hungarian oil production company. The produced water was pre-purified by the local wastewater treatment technology including chemical destabilization and sand filtration. By the application of the installed technology, the current emission limit values are achieved, but the organoleptic properties of the treated wastewater (such as smell, color, and turbidity) suggest the presence of remaining oily contaminants in significant amounts. Moreover, higher purification efficiency is expected to be required in the future to ensure environmental sustainability. The pretreated wastewater was characterized by conductivity, pH, turbidity, chemical oxygen demand (COD), and extractable oil content (TOG/TPH) measurements (Table 1).

**Table 1:** Measured properties of the investigated produced water after the local pretreatment

Turbidity (NTU)	pH	Conductivity (mS cm^−1^)	COD (mg L^−1^)	TOG/TPH (mg L^−1^)
44.2 ± 0.5	7.65 ± 0.05	5.1 ± 0.05	927 ± 10	28 ± 2

### Ozonation

When the produced water was pre-ozonated (before membrane filtration), the ozone was generated from clean oxygen (*Messer*; 3.5) by a flow-type ozone generator (*BMT 802X, Germany*), and it was bubbled through a glass diffuser into a batch reactor (*V* = 500 mL) for 2 min. The ozone concentration of the inlet was 14 ± 1 mg L^−1^, the gas flow rate was 1 L min^−1^, and the absorbances of inlet and outlet were measured in every 30 s with a UV spectrophotometer (*WPA Biowave II, UK*) at *λ* = 254 nm to calculate the absorbed ozone dose (ε_ozone, λ = 254 nm_ = 3000 M^−1^ cm^−1^), which was 28 ± 2 mg L^−1^, after 2 min. The remaining dissolved ozone was purged out by oxygen after the treatments (*t* = 5 min) to avoid the possible damage of the polymeric membranes during the subsequent membrane filtration experiments. The effects of pre-ozonation on the size distribution of oil droplets and on zeta potential were investigated by dynamic light scattering measurements (Malvern ZetaSizer Nano ZS, UK; *λ* = 633 nm, *T* = 25 ± 0.1 °C).

### Membrane filtration

Membrane filtration experiments were carried out in a magnetically stirred dead-end reactor (*Millipore XFUF07601, USA*) equipped with a circular (filtration area: 37.4 cm^2^) PES, PVDF, or PTFE ultrafilter (UF) membranes (New Logic Research INC, USA) or two different kinds of nanomaterial-covered PVDF membranes (MWCO = 100 kDa, for each membrane). The PTFE membranes were preconditioned before the filtration experiments by soaking them in acetone (Spektrum 3D, 99.5% purity) for 1 h to make them hydrophilic, since in their original form, they are hydrophobic and they can be used for water filtration only after this (or other kind of) conditioning procedure (Hong et al. 2003). In the case of commercial PES, PVDF, and modified PVDF membranes, simple water soaking was applied before the experiments. During the filtration experiments, 0.1 MPa transmembrane pressure and 5.83 s^−1^ (350 rpm) stirring speed were applied. In all cases, 200 mL permeate was filtered from the initial 250 mL volumes (volume reduction ratio, VRR = 5).

### Modification of commercial PVDF membranes with nanomaterials

The surfaces of commercial PVDF membranes were modified in some cases by covering them with titanium dioxide nanoparticles (TiO_2_; Aeroxide P25, Germany, *d* = 25–39 nm, a^S^_BET_ = 50.6 m^2^ g^−1^) or carbon nanotube-containing (CNT; Nanothinx NTX1 multi-walled carbon nanotube, Greece, l ≥ 10 μm; *d* = 15–35 nm) TiO_2_/CNT nanocomposites (containing 1 wt% of CNT). The nanomaterials were suspended in 2-propanol (m_nanomaterial_ = 40 mg, V_2-propanol_ = 100 mL) by ultrasonic homogenization (Hielscher UP200S, Germany) at 25 °C for 2 min (maximal amplitude and cycle were applied). Then for the physical deposition of the nanomaterials, the suspension was filtered through the membrane, applying 0.3 MPa transmembrane pressure, then it was dried in air at room temperature (final nanomaterial coverage was ~ 1.0 mg cm^−2^). This physical deposition resulted in the durable immobilization of the nanoparticles in our experimental conditions, which was proved by an additional experiment: a coated membrane was placed into the membrane reactor filled with pure water, which was continuously and intensively stirred for 12 h. During this experiment, no significant particle leaching was observed, which was reinforced by mass measurements of the dried membrane and turbidity measurements of the stirred water.

### Determination of purification efficiency

The purification efficiency of membrane filtration was determined by measuring the turbidity, chemical oxygen demand (COD), and extractable oil content (TOG/TPH). Turbidity values were measured with a Hach 2100 N-type nephelometric turbidity meter. COD values were measured by a standard method based on potassium dichromate oxidation using standard test tubes (*Hanna Instruments, USA*), a *Lovibond ET 108*-type digester (for 2 h, at *T* = 150 °C), and a *Lovibond COD Vario-*type photometer*.* Extractable oil content was measured by a *Wilks InfraCal TOG/TPH*-type analyzer, using hexane as extracting solvent. The purification efficiencies (R) were determined as:

1 $$ \mathrm{R}=\left(1-\frac{a}{a_0}\right)\cdotp 100\% $$

where *a*_*0*_ is either the turbidity, COD, or TOG/TPH value of the feed, while *a* indicates the value of the permeate.

### Calculation of different filtration resistances and fouling resistance abilities

The membrane resistance (*R*_*M*_) was calculated as:

2 $$ {\mathrm{R}}_M=\frac{\Delta  p}{J_W{\eta}_W}\ \left[{m}^{-1}\right] $$

where Δ*p* is the transmembrane pressure [Pa], *J*_*W*_ is the water flux of the clean membrane [m^3^ m^−2^ s^−1^], and *η*_*W*_ is the viscosity of the water [Pa s].

The irreversible resistance (*R*_*Irrev*_) was determined by remeasuring the water flux on the used membrane after the filtration, followed by a purification step (intensive rinsing with distilled water):

3 $$ {\mathrm{R}}_{Irrev}=\frac{\Delta  p}{J_{WA}{\eta}_W}-{R}_M\ \left[{m}^{-1}\right] $$

where *J*_*WA*_ is the water flux after the cleaning procedure.

The reversible resistance (*R*_*Rev*_ – caused by weakly adhered contaminants and concentration polarization layer) can be calculated as:

4 $$ {\mathrm{R}}_{Rev}=\frac{\Delta  p}{J_c{\eta}_{WW}}-{R}_{Irrev}-{R}_M\ \left[{m}^{-1}\right] $$

where *J*_*c*_ is the flux at the end of the filtration and *η*_*ww*_ is the viscosity of the wastewater. The total resistance (*R*_*T*_) can be calculated as:

5 $$ {\mathrm{R}}_T={R}_M+{R}_{Irrev}+{R}_{Rev}\ \left[{m}^{-1}\right] $$

To evaluate the fouling resistance ability of the membranes, the flux decay ratio (*FDR*) and the flux recovery ratio (*FRR*) were also calculated:

6 $$ \mathrm{FDR}=\frac{J_W- Jc}{J_W}\ 100\% $$

7 $$ \mathrm{FRR}=\frac{J_{WA}}{J_W}\ 100\% $$

where *J*_*W*_ is the water flux of the clean membrane, *J*_*c*_ is the flux at the end of the filtration of the oily wastewater (at VRR = 5), and *J*_*WA*_ is the water flux after the cleaning procedure.

### Membrane surface characterization by contact angle measurements

For the description of the hydrophilicity of the investigated membranes, the contact angles – formed between the membrane surfaces and distilled water droplets – were measured using the sessile drop method (*DataPhysics Contact Angle System OCA15Pro, Germany*) at room temperature. Ten microliters of distilled water were carefully dropped onto the surface, and contact angles were immediately measured, within 3 s. The measurements were repeated five times, and the average values were calculated. In the case of the PES and PVDF membranes, the contact angles were determined to be 55.9 ± 0.8° and 57.2 ± 0.6°, respectively. In the case of nanomaterial (both TiO_2_ and TiO_2_/CNT)-covered membranes, the dropped water spread immediately on the surface, so the contact angles could not be measured (they can be regarded as zero). The PTFE membrane was hydrophobic in its original form as the contact angle was 105.5 ± 2.5°, but after acetone conditioning, the hydrophilicity cannot be determined as the surface was wet.

### Surface analysis of membranes

Surface morphology characterization and semiquantitative chemical analysis of the membranes were performed – in some cases – with a HITACHI S-4700 Type II cold field emission scanning electron microscope (SEM) operated at 10 or 20 kV accelerating voltages, by using the integrated secondary electron detector or Röntec QX2 EDS detector.

## Results and discussion

### Effects of pre-ozonation on the produced water

**Fig. 1:**
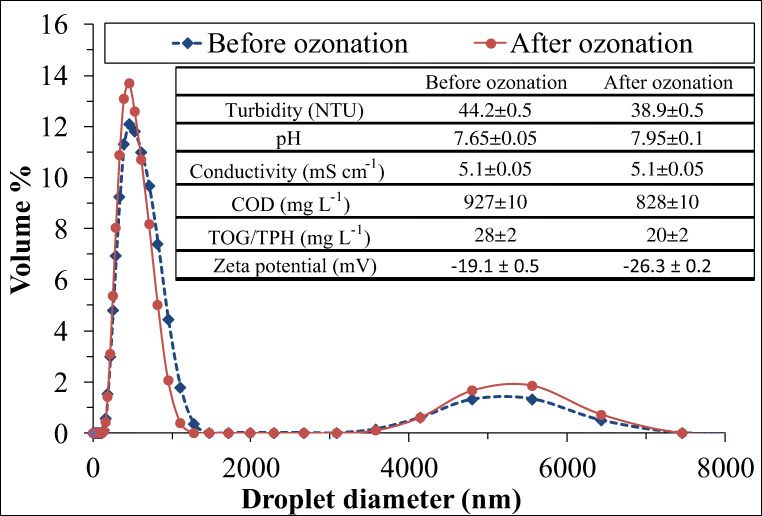
Effects of pre-ozonation on the conductivity, pH, turbidity, chemical oxygen demand (COD), extractable oil content (TOG/TPH), zeta potential, and size distribution of the produced water

Firstly, the effects of pre-ozonation on the conductivity, pH, turbidity, chemical oxygen demand (COD), extractable oil content (TOG/TPH), zeta potential, and size distribution of the produced water were investigated. On the basis of our previous study (Veréb et al. 2018b), just the very brief pre-ozonation of oil-in-water emulsions leads to significantly increased flux during the ultrafiltration; therefore, only 2-min-long pre-ozonation was applied, which resulted in 28 ± 2 mg L^−1^ of absorbed ozone dose. The determined changes in the produced water’s characteristics are presented in Fig. 1.

While the conductivity and pH did not change significantly by this short treatment, the COD and TOG/TPH values decreased notably – from 927 mg L^−1^ to 828 mg L^−1^ and from 28 mg L^−1^ to 20 mg L^−1^, respectively – due to the oxidation, and the negative surface charge of the oil droplets increased significantly as it is indicated by the zeta potential values: it increased from − 19.1 mV to − 26.3 mV after ozonation. The dynamic light scattering measurements indicated polydisperse size distribution with two volume maximums at 460 and 5560 nm. The applied short pre-ozonation did not cause important changes in the size distribution, but interestingly, both the two well-known ozonation-related effects can be observed in Fig. 1: the fragmentation effect (Veréb et al. 2018b) in the case of nanoscaled droplets (caused by the high oxidation capacity of ozone) and the microflocculation effect in the case of micro-sized droplets. The latter effect can be related to many different explanations as it is well described in the literature (Jekel 1994):

- Ozone may release/oxidize Fe^2+^ or Mn^2+^ ions of organometallic complexes, leading to coagulation by hydroxide precipitates.
- Ozonation may induce a partial polymerization of dissolved organics, forming polyelectrolytes.
- The loss of CO_2_ can induce CaCO_3_ precipitation and particle aggregation.

This latter explanation may also explain the slight increase in the pH value, which was observed during our experiments.

### Fluxes of different membranes with and without pre-ozonation

**Fig. 2:**
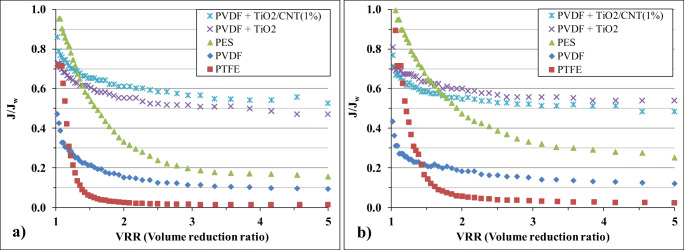
Measured relative flux curves in the case of (**a**) not pre-ozonized and (**b**) pre-ozonized produced waters

Membrane filtration experiments were carried with the investigated 5 different membranes with and without pre-ozonation. During the filtration, the fluxes were measured continuously until the volume reduction ratio was 5. The measured flux curves are presented in Fig. 2.

In the case of the absence of pre-ozonation (Fig. 2a), by applying any of the three commercial membranes – PES, PVDF, and PTFE – quick and significant flux reduction was observed, which resulted in negligible stabilized flux values (at VRR = 5): 104, 121, and 62 L m^−2^ h^−1^, respectively. Nevertheless, in the case of nanomaterial-modified PVDF membranes, much lower flux reductions were observed both in the case of TiO_2_ and TiO_2_/CNT impregnations. The stabilized fluxes were more than 2.5 times higher compared to the uncoated commercial PVDF membrane: 326 and 301 L m^−2^ h^−1^, in the case of TiO_2_ and TiO_2_/CNT coatings, respectively. Considering the low transmembrane pressure (0.1 MPa), these flux values are outstanding compared to the ones in the literature. Abadi et al. reported 200–250 L m^−2^ h^−1^ achievable fluxes by using a ceramic microfilter membrane at the same transmembrane pressure (0.1 MPa) for the purification of similarly contaminated (26 mg L^−1^) oily wastewater (Abadi et al. 2011). In the publication of Yi et al., much lower flux (~ 40 L m^−2^ h^−1^ at 0.1 MPa transmembrane pressure; no available data on the oil concentration) was demonstrated in the case of TiO_2_/Al_2_O_3_-modified PVDF membranes (Yi et al. 2011). Saki et al. also published much lower achievable fluxes (in the case of a heavily oil-contaminated water) by using their PSF/PEI/CaCO_3_ nanocomposite UF membranes: fluxes were 28, 26, 32, and 98 L m^−2^ h^−1^ for the membranes with 1, 2, 5, and 10 wt% CaCO_3_ nanoparticle loadings, respectively, at 2 bar pressure (Saki and Uzal 2018). Our results confirm the significant advantages of the used highly hydrophilic nanomaterial coating in the case of real oily wastewaters (produced waters), as it inhibited the adherence of the oil droplets to the surface, preventing the formation of a hydrophobic water barrier layer and the fouling of the pores.

By the application of pre-ozonation, significant flux increasing effects were observed in the case of commercial membranes (Fig. 2b): the stabilized flux values were 177, 155, and 125 L m^−2^ h^−1^ by using the PES, PVDF, and PTFE membranes, respectively. These fluxes mean 70, 28, and 102% increases, respectively, which can be interpreted as significant improvements, but these values are still far lower than the achievable fluxes by using the nanomaterial-covered membranes. Nevertheless, the fluxes slightly increased after pre-ozonation as well when TiO_2_ and TiO_2_/CNT-modified membranes were used: these values were 362 and 339 L m^−2^ h^−1^, respectively (11 and 13% increase).

On the basis of the measured fluxes, it can be interpreted that even the more negatively charged oil droplets (resulted by the pre-ozonation) can significantly decrease the adhesion of the oil droplets on the membrane surfaces, but the hydrophilicity of the surface plays a more significant role in the adherence of these hydrophobic contaminants than the electrostatic forces: the hydrophilic coverages resulted in much higher fluxes, while pre-ozonation caused just mild further increases.

### Filtration resistances

**Fig. 3:**
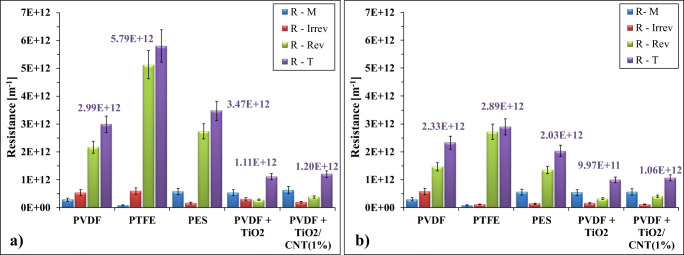
Calculated membrane, reversible, irreversible, and total filtration resistances in the case of (**a**) not pre-ozonized and (**b**) pre-ozonized produced waters

The calculated different filtration resistances (membrane, reversible, irreversible, and total resistances) are presented in Fig. 3.

In general, pre-ozonation reduced the total filtration resistance in the case of each membrane, although the degree of this effect varies greatly, depending on the membrane. On one hand, the most significant total filtration resistance reduction was determined in the case of the PTFE membrane (both irreversible and reversible resistances were reduced), but on the other hand, the highest total filtration resistance was also observed in the case of this membrane after pre-ozonation (Fig. 3b). Although the PTFE membrane had the lowest membrane resistance – due to the pre-conditioning procedure with acetone – but the high initial flux can cause a rapid buildup of a significant concentration polarization layer and the intense coalescence of the hydrophobic contaminants. Furthermore, the presence of acetone also contributes to the adhesion of hydrophobic oil droplets on the surface. Overall, these effects resulted in the highest reversible and irreversible resistances of this series in the case of the PTFE membrane. Pre-ozonation was able to significantly reduce these mechanisms by the increased electrostatic repulsive forces, resulting significantly lower reversible and irreversible resistances, but for the separation of oily contaminants the acetone pre-conditioned PTFE membrane cannot be recommended neither with nor without pre-ozonation.

A significant reduction of the total filtration resistance was observed also in the case of PES and PVDF membranes, which mainly originated from the reduced reversible resistances. However, the lowest total filtration resistances were observed during the utilization of TiO_2_ and TiO_2_/CNT-covered membranes, and the reversible and irreversible resistances were very low too. Pre-ozonation further reduced slightly the total filtration resistances, which were most likely originated from the reduced irreversible resistances (Fig. 3a, b). Overall, the filtration resistances also proved the outstanding advantages of nanomaterial-modified membranes.

### Fouling resistance ability of the membranes

**Fig. 4:**
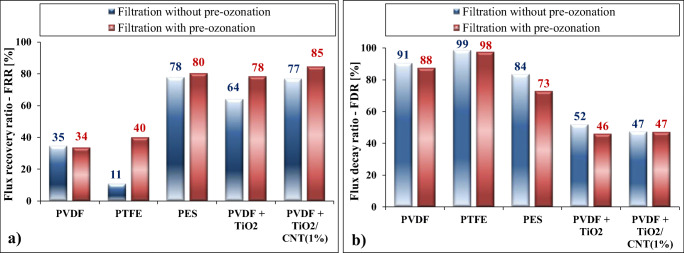
Calculated flux recovery ratios (FRR) and flux decay ratios (FDR) in the case of (**a**) not pre-ozonized and (**b**) pre-ozonized produced waters

To determine the fouling resistance ability of the membranes, the flux decay ratios (*FDR*) and the flux recovery ratios (*FRR*) were calculated, both in the presence and in the absence of pre-ozonation. The results are presented in Fig. 4.

FRR values indicate the percentage recovery of the original water flux of the clean membrane after its utilization and purification by water rinsing, so higher values are beneficial and indicate better antifouling property. As it is presented in Fig. 4a, the application of pre-ozonation always increased the FRR values. This means better cleanability of the membranes, which can be explained by the more negative zeta-potential values after pre-ozonation, which increased the electrostatic repulsive forces between the droplets themselves – resulting in reduced coalescence – and between the droplets and membrane surfaces, making the adherence of the contaminants to the surface less likely. The highest FRR value was determined in the case of the TiO_2_/CNT-covered membrane, when it was used for the filtration of the pre-ozonized wastewater, but the PES membrane and the TiO_2_-covered PVDF membranes also had excellent FRR values.

As FDR values indicate the percentage flux decays during the filtration, the lower values are favorable; therefore, the nanomaterial-covered membranes were the most beneficial in relation to these values (Fig. 4b). A general, minor beneficial effect of pre-ozonation was also observed in accordance with the FDR values.

### Photocatalytic cleaning of the contaminated nanomaterial-coated membranes

**Fig. 5:**
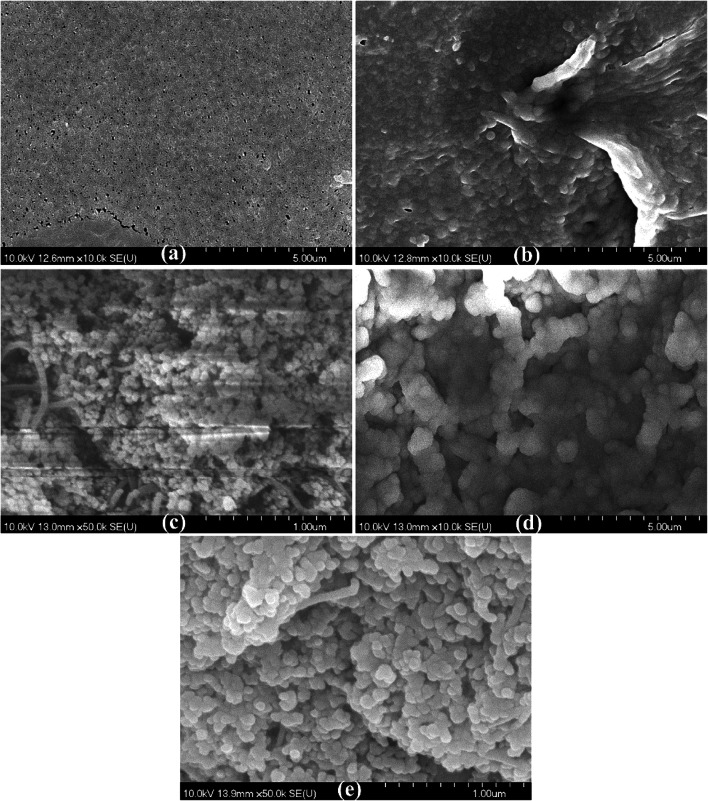
SEM micrographs of the (**a**) neat PVDF membrane, (**b**) used (contaminated) PVDF membrane, (**c**) clean TiO_2_/CNT-coated membrane, (**d**) TiO_2_/CNT-coated membrane after use (contaminated), and (**e**) the contaminated TiO_2_/CNT-coated membrane after its photocatalytic purification

**Fig. 6:**
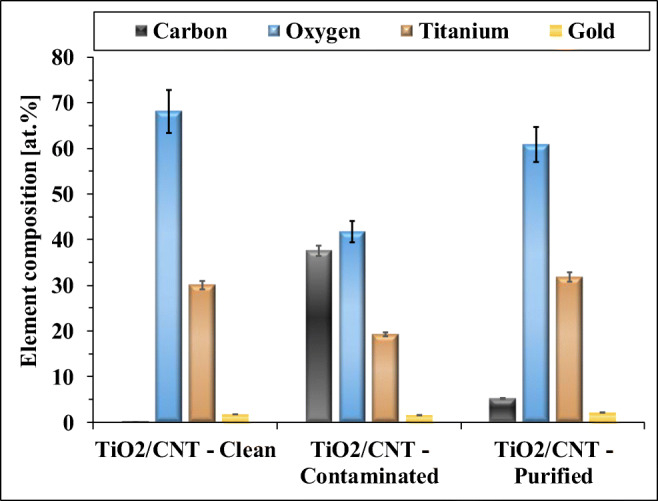
Results of EDX elemental analysis of the different membranes (The presence of gold is due to the necessary gold-coating procedure of the samples, which was applied before the measurements)

In the case of the photocatalyst (both the TiO_2_ and the TiO_2_/CNT) covered membranes, after a 12-h–long UV irradiation (Lightech, 10 W, λ_max_ ∼ 365 nm, UV intensity: 24 mW m^−2^), the original water flux was completely recovered, due to the photocatalytic degradation of the remaining contaminants. Control experiments were also carried out, and without UV light, no notable change of the flux was measured, while the UV irradiation of the oil-contaminated, but not nanomaterial-coated membrane, did not result in any measurable flux recovery either. In addition, some micrographs were taken by scanning electron microscopy (Fig. 5), and elemental analysis (Fig. 6) were also carried out for the TiO_2_/CNT-coated, the oil-contaminated, and the photocatalytically purified membranes. The micrographs (Fig. 5) show that in the case of uncoated membrane, a thick contaminant layer formed, while the TiO_2_/CNT coating significantly reduced the amount of accumulated contaminants. The successful photocatalytic purification was confirmed by the micrographs (Fig. 5), and the elemental analysis of the membranes also proved the significant reduction of carbon content by photocatalytic purification (Fig. 6).

### Purification efficiencies

**Fig. 7:**
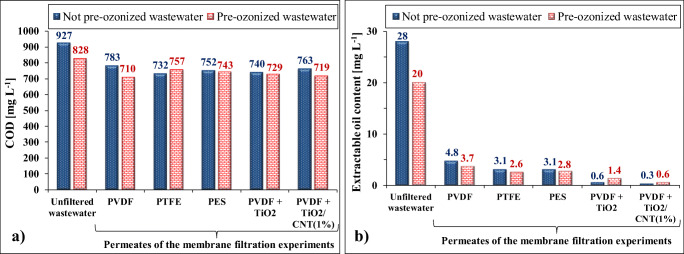
**a** Chemical oxygen demand (COD) and **b** extractable oil content of the original/ozonized wastewaters and the permeates, both in the case of the presence and absence of pre-ozonation

Purification efficiencies of membrane filtrations were always determined by measuring the COD, turbidity, and TOG/TPH values of the permeates. In relation to the COD values (Fig. 7a), it can be estimated that pre-ozonation resulted in a 12% decrease, while membrane filtration resulted in a 16–23% decrease, but there was not any significant difference by using the different membranes or by the presence/absence of pre-ozonation. The measured high COD values of the permeates indicate high amount of dissolved oxidizable (organic and/or inorganic) compounds in the wastewater. Turbidity values always indicated > 99% purification efficiency. The extractable oil contents (TOG/TPH) of the different samples are presented in Fig. 7b.

Extractable oil content significantly decreased even by the short pre-ozonation (from 28 to 20 mg L^−1^), and after the membrane filtration, 83–91% purification efficiencies were determined when the commercial membranes were used. During the application of the nanomaterial-covered membranes, these values were 95–99%. Interestingly, in the case of commercial membranes, pre-ozonation resulted in slightly higher oil elimination efficiencies, which can be explained by the increased electrostatic repulsive force between the membrane and the contaminants. Nevertheless, the outstanding oil separation efficiency of the nanomaterial-covered membranes cannot be enhanced further by the short pre-ozonation; moreover, slightly lower efficiencies were determined in these cases.

Overall, in relation to the purification efficiency, the nanomaterial-modified membranes were the most beneficial, especially without pre-ozonation, resulting in excellent oil removal efficiency.

## Conclusions

Pre-ozonation resulted in the reduction of total filtration resistance in all cases (especially in the case of the commercial ultrafilter membranes), independently from the membrane material. However, far from the lowest filtration resistances were measured in the case of nanomaterial (TiO_2_ and TiO_2_/CNT)-modified membranes, and in this case, pre-ozonation caused only a slight further reduction of the total filtration resistance.

In relation to the flux recovery ratio (FRR), the utilization of TiO_2_/CNT-covered membrane combined with pre-ozonation was the most beneficial, but the nanomaterial-covered membranes and PES membrane also showed great values. Ozone pretreatment resulted in increased FRR values (except for the PVDF membrane), proving that better antifouling properties can be achieved by its application.

The elimination of extractable oil content was 83–91% in the case of commercial membranes and 95–99% for nanomaterial-coated membranes. Pre-ozonation caused a slight decrease in purification efficiency when the modified membranes were used.

The highest fluxes were also measured in the case of nanomaterial-coated membranes. Without pre-ozonation, 326 and 301 L m^−2^ h^−1^ values were measured, while by applying pre-ozonation, 362 and 339 L m^−2^ h^−1^ fluxes were achieved in the case of TiO_2_ and TiO_2_/CNT coatings, respectively.

Overall, the utilization of nanomaterial-modified membranes was more beneficial than pre-ozonation in the case of the investigated oily wastewater. The combination of pre-ozonation with filtration using nanomaterial-modified membranes was also advantageous concerning the filtration properties such as the flux, filtration resistances, and antifouling properties.
